# Prevalence of operating room occupational health cluster: a systematic review and meta-analysis

**DOI:** 10.3389/fpubh.2026.1792542

**Published:** 2026-05-26

**Authors:** Shuai Zhang, Hongchuang Ji, Zhaohao He, Liurong Cheng, Jiaqi Li

**Affiliations:** 1 Department of Nursing, Hainan General Hospital, Haikou, China; 2 Operation Room, Hainan General Hospital, Haikou, China

**Keywords:** healthcare personnel, job burnout, meta-analysis, musculoskeletal injury, occupational exposure, operating room, systematic review

## Abstract

**Objective:**

To assess the global prevalence of occupational health impairments among operating room personnel (Operating Room Occupational Health Cluster) and provide evidence for targeted health interventions.

**Methods:**

We conducted computerized searches of PubMed, Embase, Web of Science, CINAHL, and the Cochrane Library for cross-sectional and cohort studies on occupational injuries among operating room personnel, spanning from database inception to January 1, 2026. Two researchers independently screened studies, extracted data, and assessed quality. Meta-analysis was performed using RevMan 5.4, with logit-transformed raw rates pooled using fixed- or random-effects models based on heterogeneity.

**Results:**

Forty studies with 23,816 participants were included. Meta-analysis revealed: (1) Musculoskeletal injuries: Overall prevalence was 56.52% [95% CI (47.64, 65.03%)], with lumbar pain (76.13%), shoulder/neck pain (63.14%), and knee pain (58.91%) most common. (2) Job Burnout: Prevalence was 52.38% [95% CI (42.86, 65.15%)], with emotional exhaustion at 46.24%. (3) Occupational Exposure: Blood and body fluid exposure occurred at 45.05%, surgical smoke exposure at 69.71%, and sharp object/needle stick injuries at 16.07 and 25.51%, respectively. (4) Mental Health: Depression prevalence was 59.14% [95% CI (54.24, 68.78%)], and anxiety at 36.31%. Descriptive analysis indicated high levels of fatigue among operating room personnel.

**Conclusion:**

The prevalence of Operating Room Occupational is high, with musculoskeletal pain, depression, and occupational exposure being particularly severe. Healthcare management should prioritize the physical and mental health of operating room personnel, optimize workflows, and strengthen occupational protection measures.

**Systematic review registration:**

https://www.crd.york.ac.uk/prospero/display_record.php?ID=CRD420251275100, CRD420251275100.

## 1 Introduction

Operating rooms, as the primary setting for critical care, are characterized by high-intensity, high-pressure, and high-risk environments. Operating room personnel face continuous exposure to occupational hazards such as surgical instrument noise, ionizing radiation, and chemical disinfectants, while simultaneously managing demanding tasks like precise surgical coordination, emergency response to unforeseen events, and prolonged ergonomic strain from periods of standing and bending. These factors contribute to persistent physical and mental strain (1, 2). To enhance conceptual clarity, this study operationalizes this construct as the “Operating Room Occupational Health Cluster”. It is defined as a multidimensional symptom cluster encompassing musculoskeletal disorders, professional burnout, and psychological distress (specifically anxiety and depression), all of which are systematically linked to the unique stressors and environmental exposures inherent to the operating suite.

The physical and mental health of operating room personnel not only affects their quality of life but also directly influences the quality of nursing services, surgical coordination, and medical safety. Research suggests that occupational burnout can decrease work motivation and increase error rates among nurses (3). Musculoskeletal disorders, common among operating room nurses, often result in symptoms such as lumbar, cervical, and shoulder pain, and in severe cases, may lead to a loss of labor capacity. Psychological conditions, such as anxiety and depression, exacerbate occupational fatigue, elevate turnover risk, and contribute to the ongoing global nursing shortage (4, 5). Therefore, accurately assessing the prevalence of Operating Room Occupational Health Cluster and identifying its epidemiological characteristics and contributing factors are crucial for developing targeted prevention and intervention strategies to ensure workforce stability and preserve healthcare quality.

In recent years, researchers worldwide have conducted numerous cross-sectional studies on the individual indicators within the operating room, such as burnout, musculoskeletal disorders, and psychological issues. However, these findings exhibit considerable heterogeneity, arising in part from differences in diagnostic tools and evaluation criteria. For example, assessments of occupational burnout use various versions of the Maslach Burnout Inventory (MBI), while surveys on musculoskeletal disorders employ instruments like the Nordic Questionnaire and visual analog scales (6). Additionally, variations in geographical location, age distribution, and clinical experience among study populations contribute to inconsistent results. Many studies remain limited to specific regions and lacking a comprehensive global perspective, furthermore, the prevalence of single-center, small-sample designs restrict the generalizability of their conclusions. To data, no systematic review or meta-analysis has synthesized the evidence through the integrated construct of the operating room occupation health cluster, preventing a thorough and precise synthesis of available evidence. This gap hampers the provision of reliable data to inform clinical interventions and policy development.

Building on these considerations, this study utilizes systematic review and meta-analysis methodologies to address these limitations. A comprehensive global literature search was conducted focusing on the core components of the operating room occupation health cluster, specifically occupational burnout, musculoskeletal disorders (MSDs), anxiety, and depression. The goal is to identify variations in prevalence rates and sources of heterogeneity across different populations, regions, and research methodologies, thereby addressing the limitations of individual studies. This approach will provide high-quality, evidence-based support for the development of targeted clinical interventions, the optimization of the operating room nursing environment, and the protection of nurses’ physical and mental health.

## 2 Methods

This study strictly adheres to the Preferred Reporting Items for Systematic Reviews and Meta-Analyses (PRISMA) guidelines. The study protocol was pre-registered in PROSPERO (CRD420251275100).

### 2.1 Inclusion and exclusion criteria

#### 2.1.1 Inclusion criteria

(1) Study type: Cross-sectional studies, cohort studies, case–control studies, or other study types providing relevant data. While multiple study designs were eligible, identified literature primarily consisted of cross-sectional and cohort designs; (2) Study population: Operating room healthcare personnel (including nurses, physicians, anesthesiologists, etc.); (3) Primary outcomes: Core indicators included in the meta-analysis consist of musculoskeletal injuries, job burnout, occupational exposure (e.g., needle sticks, smoke), and mental health conditions (anxiety, depression). Secondary outcome: Indicators such as dry eye syndrome, metabolic diseases, and varicose veins were also included in the search; however, due to insufficient data or limited study counts, these were synthesized via descriptive analysis rather than meta-analysis.

#### 2.1.2 Exclusion criteria

(1) Duplicate publications; (2) Literature such as reviews, case reports, and conference abstracts; (3) Literature with incomplete data availability; (4) Low-quality literature.

### 2.2 Search strategy

A computerized search was conducted across multiple databases, including PubMed, Embase, Web of Science, CINAHL, and the Cochrane Library, to identify studies investigating the prevalence of occupational injuries among operating room nurses. The search spanned from the inception of each database up to January 1, 2026. A combined search strategy was employed, using both subject headings and free-text terms, supplemented by manual backtracking methods. Key search terms included: “operating rooms/operating room personnel,” “cumulative trauma disorders,” “low back pain,” “intervertebral disc displacement,” “dry eye syndrome,” “sleep initiation and maintenance disorders,” “metabolic diseases,” “varicose veins,” “urinary calculi,” “urinary tract infections,” “occupational exposure,” “burnout, psychological,” “burnout, professional,” “anxiety,” “depression,” “post-traumatic stress disorder,” “obsessive-compulsive disorder” and “chronic fatigue syndrome.” Two researchers independently screened and extracted relevant data according to the inclusion and exclusion criteria. Disagreements were resolved through consultation with a third researcher. An example of the search strategy used in PubMed is provided in Box 1.

Box 1 **PubMed search formula.**

1# “operating rooms”[Mesh] OR “nurses, operating room”[Mesh] OR “surgeons”[Mesh] OR “anesthesiologists”[Mesh] OR “nurse anesthetists”[Mesh] OR “perioperative nursing”[Mesh] OR “perioperative care” [Mesh]

2# “operating room personnel”[tiab] OR “operating room staff”[tiab] OR “operating theater staff”[tiab] OR “operating theatre staff”[tiab] OR “perioperative staff”[tiab] OR “surgical team”[tiab] OR “scrub nurse*” OR “circulating nurse*”[tiab] OR “anesthetist*”[tiab])

3# 1# AND 2#

4# “occupational diseases” [Mesh] OR “burnout, professional”[Mesh] OR “musculoskeletal diseases”[Mesh] OR “occupational exposure”[Mesh] OR “occupational stress”[Mesh]

5# “syndrome”[tiab] OR “occupational health”[tiab] OR “burnout”[tiab] OR “musculoskeletal disorder*”[tiab] OR “fatigue”[tiab] OR “incidence”[tiab] OR “prevalence”[tiab] OR “frequency”[tiab])

6# 4# AND 5#

7# 3 AND 6#

### 2.3 Screening and data extraction

Two evaluators independently screened the literature and extracted data according to the predefined inclusion and exclusion criteria. Any disagreements during this process were resolved through mutual discussion or adjudication by a third senior researcher to ensure objectivity and consistency. The screening process involved an initial review of article titles, followed by further assessment of abstracts and full-text articles for those meeting the inclusion criteria. The extracted data primarily included the following: first author, publication year, country, study design, sample source, assessment tool, sample size, and the prevalence of Operating Room Occupational Health Cluster.

### 2.4 Quality evaluation

This study employed the 2016 JBI Cross-Sectional Study Quality Assessment Criteria, which consists of eight core items. These criteria systematically evaluate the methodological quality and risk of bias across eight dimensions: sample inclusion criteria definition, description of study subjects and setting, validity and reliability of exposure measurement, objectivity in disease/status measurement, identification and management of confounding factors, validity and reliability of outcome measurement, and appropriateness of statistical analysis. Each criterion was assessed using a four-point scale (Yes/No/Unclear/Not Applicable). Studies were categorized into three quality levels based on the proportion of criteria rated “Yes”: High (≥70%), Moderate (50–69%), and Low (<50%). For case–control and cohort studies, the Newcastle-Ottawa Scale was used to assess study quality, evaluating three dimensions: selection of the study population, comparability of groups, and outcome measurement. The maximum total score on this scale is 9 points, with higher scores indicating better study quality and lower risk of bias. Two researchers independently assessed the quality of each study, and any disagreements were resolved through mutual discussion or consultation with a third researcher.

### 2.5 Statistical analysis

This study utilized RevMan 5.4 software for statistical analysis, with the prevalence of Operating Room Occupational Health Cluster serving as the effect size with 95% confidence intervals (*CIs*) calculated. To ensure normality, raw prevalence rates were transformed using the Logit transformation method. The pooled estimates and corresponding 95%*CI* were then back transformed to original proportions (*p*) using the inverse logit formula: p=exp.(L)/1+exp.(L) , where *L* denotes the pooled estimate on the logit scale. The transformation ensures that both the derived prevalence and the limits of the confidence intervals remain within the range of 0–1.

Study consistency was assessed using the Cochrane *Q* test and *I*^2^ statistics If *I*^2^ ≥ 50% and *p* ≤ 0.10, indicating significant heterogeneity, a random-effects model was used; otherwise, a fixed-effect model was employed.

To address extreme heterogeneity (*I*^2^ ranging from 88 to 100%), the following strategies were implemented: (1) Sensitivity analysis: A stepwise exclusion method was used to evaluate the impact of individual studies on the stability of pooled results. For instance, specific studies (e.g., Simonsen et al. or Bos et al.) were excluded *post hoc* to explore sources of variability and verify the robustness of the findings. (2) Subgroup analysis: Analyses were stratified by publication year (pre-2010 vs. post-2010) or study population (nurses vs. anesthesiologists). (3) Precision trap clarification: In analyses of burnout and anxiety, large-scale studies (e.g., Almodibeg et al. and Che et al.) contributed disproportionately low standard errors. This creates a precision trap where the *I*^2^ index becomes hyper-sensitive to minor deviations in effect sizes, artificially inflating statistical heterogeneity despite high internal precision.

Given that fewer than 10 studies were included in each sub-analysis, publication bias assessments (e.g., Begg’s or Egger’s tests) were not conducted to avoid misinterpretation due to insufficient statistical power, per Cochrane Collaboration guidelines.

## 3 Results

### 3.1 Search results

**Figure 1:**
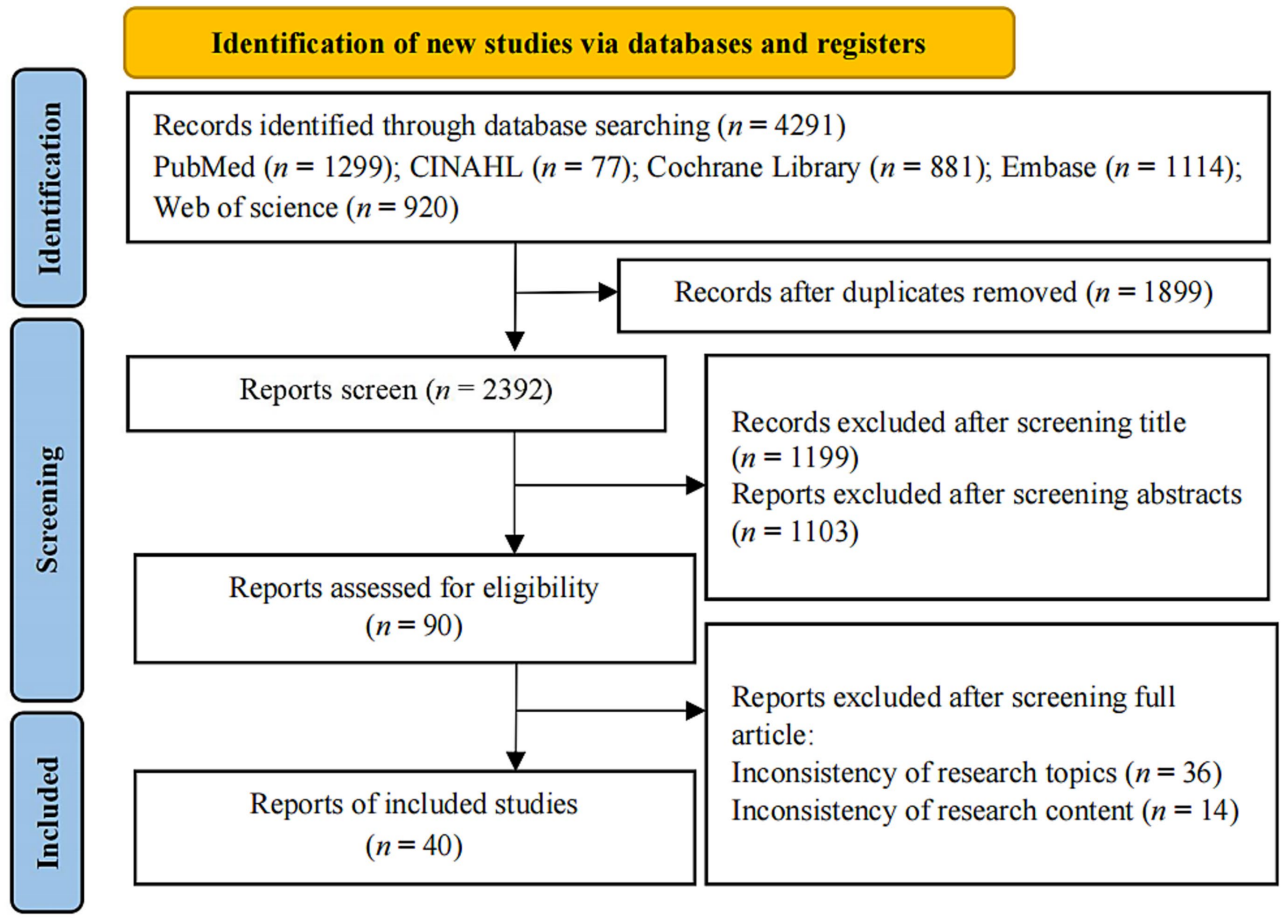
PRISMA flow diagram.

This study included 40 publications involving a total of 23,816 operating room personnel. Among these, 38 were cross-sectional studies (7–44) and 2 were cohort studies (26, 45). The primary manifestations of Operating Room Occupational Health Cluster identified in the studies included muscle injury, occupational exposure, occupational burnout, anxiety, depression, post-traumatic stress disorder, fatigue, and low self-esteem. Three studies (18, 19, 21) specifically addressed the impact of the COVID-19 pandemic. The study participants comprised operating room nurses, surgeons, anesthesiologists, and technicians. The flowchart of the literature search process is shown in Figure 1, and the general characteristics of the included studies are summarized in Table 1.

**Table 1:** General information on included literature (*n* = 40).

Author	Country	Population	Research type	Age	Sample	Female	Outcome
Liu et al. 2025 (7)	China	Nurse	Cross-sectional study	34.62	744	289	Job burnout
Gjini et al. 2025 (8)	Albania	Personnel	Cross-sectional study	24 ~ 66	101	58	Occupational exposure
Fu et al. 2025 (9)	China	Nurse	Cross-sectional study	/	258	68	Compassion fatigue
Li et al. 2025 (10)	China	Nurse	Cross-sectional study	21 ~ 60	60	11	Emotional exhaustion
Kim and Shin, 2025 (11)	Korea	Nurse	Cross-sectional study	26 ~ 30	150	22	Mental health
Rahmani et al. 2025 (12)	Iran	Nurse	Cross-sectional study	28.86 ± 6.15	385	96	Neurolysis
Zhai et al. 2025 (13)	China	Nurse	Cross-sectional study	21 ~ 50	386	61	Fatigue
Hasan et al. 2024 (14)	Palestine	Anesthesiologists	Cross-sectional study	/	113	80	Job burnout
Yao et al. 2024 (15)	China	Nurse	Cross-sectional study	20 ~ 55	134	/	Mental health
Rambod et al. 2024 (16)	Iran	Surgical technologists	Cross-sectional study	33.76 ± 9.50	217	67	Fatigue
Alotaibi et al. 2024 (17)	Saudi Arabia	Personnel	Cross-sectional study	/	227	126	Fatigue
Aktas et al. 2024 (18)	UK	Nurse	Cross-sectional study	43.78 ± 11.73	1,127	/	Mental health
Che et al. 2023 (19)	China	Anesthesiologists	Cross-sectional study	/	6,631	1,495	Job burnout; Mental health
Sun et al. 2021 (20)	China	Health Personnel	Cross-sectional study	/	4,526	/	Occupational exposure
Gül et al. 2021 (21)	Turkey	Nurse	Cross-sectional study	36.67 ± 7.28	192	174	Mental health
Martí-Ejarque et al. 2021 (22)	Spain	Nurse	Cross-sectional study	43.48 ± 11.18	165	12	Musculoskeletal complaints; Mental health
Yizengaw et al. 2021 (23)	Ethiopia	Personnel	Cross-sectional study	30.56 ± 4.36	394	308	Musculoskeletal complaints
Wang et al. 2021 (24)	China	Nurse	Cross-sectional study	20 ~ 57	361	66	Job burnout; Mental health
Li et al. 2021 (25)	China	Nurse	Cross-sectional study	35.41 ± 6.87	509	473	Job burnout
Li et al. 2020 (45)	China	Anesthesiologists, nurse	Cohort study	20 ~ 60	197	96	Mental health
Almodibeg and Smith, 2020 (26)	Saudi Arabia	Nurse	Cross-sectional study	/	39	/	Job burnout
Clari et al. 2019 (27)	Italy	Nurse	Cross-sectional study	48 ± 6.1	148	31	Work-related musculoskeletal disorders
Freire et al. 2016 (28)	Brazil	Anesthesiologists	Cross-sectional study	27 ~ 60	198	168	Job burnout. Mental health
Kasatpibal et al. 2016 (29)	Thailand	Nurse	Cross-sectional study	37.8 ± 9.6	1,270	109	Occupational exposure
Arsalani et al. 2016 (30)	Iran	Nurse	Cross-sectional study	/	117	/	Musculoskeletal complaints
Uğurlu et al. 2015 (31)	Turkey	Nurse and technicians	Cross-sectional study	29.3 ± 6.7	74	28	Occupational exposure
Nützi et al. 2015 (32)	Switzerland	Nurse	Cross-sectional study	39.94 ± 11.90	116	3	Musculoskeletal complaints
Markovic-Denic et al. 2015 (33)	Serbia	Personnel	Cross-sectional study	37(20 ~ 65)	160	/	Occupational exposure
Miri et al. 2015 (34)	American	Personnel	Cross-sectional study	34.9 ± 9.4	512	285	Occupational exposure
Kasatpibal et al. 2015 (44)	Thailand	Nurse	Cross-sectional study	38.4 ± 9.8	2031	156	Occupational exposure
Simonsen et al. 2012 (35)	Sweden	Nurse and assistant nurse	Cross-sectional study	/	192	0	Musculoskeletal complaints
Cutter and Jordan, 2012 (43)	United Kingdom	Surgeons and nurse	Cross-sectional study	/	315	/	Occupational exposure
Hinmikaiye et al. 2012 (36)	Nigeria	Nurse	Cross-sectional study	21 ~ 60	80	24	Musculoskeletal complaints
Moscato et al. 2010 (37)	Italy	Nurse	Cross-sectional study	36.08 ± 7.08	185	112	Musculoskeletal complaints
Choobineh et al. 2010 (38)	Iran	Nurse	Cross-sectional study	31.54 ± 8.46	375	126	Musculoskeletal complaints
Sheikhzadeh et al. 2009 (39)	American	Nurse and technicians	Cross-sectional study	43.9 ± 9.1	32	/	Musculoskeletal complaints
Bos et al. 2007 (40)	Netherlands	Nurse	Cross-sectional study	40 ± 10	381	57	Musculoskeletal complaints
Meijsen and Knibbe, 2007 (41)	Netherlands	Personnel	Cross-sectional study	36 ± 10.3	463	69	Musculoskeletal complaints
Kumari et al. 2006 (42)	India	Surgeon	Cross-sectional study	/	7	/	Occupational exposure
Diaz, 2001 (53)	American	Nurse	Cohort study	40.2	244	0	Musculoskeletal complaints

### 3.2 Quality evaluation results

The results of the literature quality assessment indicate that the overall quality of the 38 included cross-sectional studies was satisfactory. Of these, 29 studies were rated as high quality, while 9 were rated as moderate quality. All studies demonstrated strong performance (100%) in Item 1 (alignment of the sampling frame with the target population), Item 2 (appropriateness of methods used for selecting study subjects), and Item 8 (appropriateness of data analysis). However, some studies showed deficiencies in addressing confounding factors. The detailed results of the literature quality assessment are presented in Table 2. The two cohort studies, both rated as high quality, were also approved for inclusion in the analysis.

**Table 2:** Quality assessment results of cross-sectional studies (*n* = 38).

Author	①	②	③	④	⑤	⑥	⑦	⑧
Fu et al. 2025 (9)	Y	Y	Y	Y	Y	Y	Y	Y
Gjini et al. 2025 (8)	Y	Y	Y	U	Y	U	Y	Y
Kim and Shin, 2025 (11)	Y	Y	Y	Y	Y	Y	Y	Y
Li et al. 2025 (10)	Y	Y	Y	Y	Y	N	Y	Y
Liu et al. 2025 (7)	Y	Y	Y	Y	Y	Y	Y	Y
Rahmani et al. 2025 (12)	Y	Y	Y	Y	Y	Y	Y	Y
Zhai et al. 2025 (13)	Y	Y	Y	Y	Y	Y	Y	Y
Aktas et al. 2024 (18)	Y	Y	Y	Y	Y	Y	Y	Y
Alotaibi et al. 2024 (17)	Y	Y	Y	Y	Y	Y	Y	Y
Hasan et al. 2024 (14)	Y	Y	Y	Y	Y	Y	Y	Y
Rambod et al. 2024 (16)	Y	Y	Y	Y	U	N	Y	Y
Yao et al. 2024 (15)	Y	Y	U	Y	U	U	Y	Y
Che et al. 2023 (19)	Y	Y	Y	Y	Y	Y	Y	Y
Gül et al. 2021 (21)	Y	Y	Y	Y	Y	Y	Y	Y
Martí-Ejarque et al. 2021 (22)	Y	Y	Y	Y	U	U	Y	Y
Sun et al. 2021 (20)	Y	Y	U	Y	U	N	U	Y
Wang et al. 2021 (24)	Y	Y	Y	Y	Y	Y	Y	Y
Yizengaw et al. 2021 (23)	Y	Y	U	Y	Y	Y	Y	Y
Li et al. 2021 (25)	Y	Y	Y	Y	Y	Y	Y	Y
Almodibeg and Smith, 2020 (26)	Y	Y	Y	Y	U	N	Y	Y
Clari et al. 2019 (27)	Y	Y	Y	Y	Y	Y	Y	Y
Arsalani et al. 2016 (30)	Y	Y	Y	Y	Y	Y	Y	Y
Freire et al. 2016 (28)	Y	Y	Y	Y	Y	Y	Y	Y
Kasatpibal et al. 2016 (29)	Y	Y	Y	Y	Y	Y	Y	Y
Kasatpibal et al. 2015 (29)	Y	Y	Y	Y	Y	Y	Y	Y
Markovic-Denic et al. 2015 (33)	Y	Y	U	Y	U	N	U	Y
Miri et al. 2015 (34)	Y	Y	Y	Y	Y	N	Y	Y
Nützi et al. 2015 (32)	Y	Y	Y	Y	Y	Y	Y	Y
Uğurlu et al. 2015 (31)	Y	Y	Y	N	U	N	N	Y
Cutter and Jordan, 2012 (43)	Y	Y	NA	Y	Y	Y	Y	Y
Hinmikaiye et al. 2012 (36)	Y	Y	U	Y	N	N	U	Y
Simonsen et al. 2012 (35)	Y	Y	Y	Y	Y	Y	Y	Y
Choobineh et al. 2010 (38)	Y	Y	Y	Y	Y	Y	Y	Y
Moscato et al. 2010 (37)	Y	Y	U	Y	Y	Y	U	Y
Sheikhzadeh et al. 2009 (39)	Y	Y	Y	N	U	N	U	Y
Bos et al. 2007 (40)	Y	Y	Y	Y	Y	Y	Y	Y
Meijsen and Knibbe, 2007 (41)	Y	Y	Y	Y	U	N	Y	Y
Kumari et al. 2006 (42)	Y	Y	Y	N	U	N	Y	Y

The quality assessment criteria for the included studies were as follows: ① Whether the inclusion criteria for samples are clearly defined. ② Whether the study subjects and settings are described in detail. ③ Whether the measurement methods for exposure factors possess reliability and validity. ④ Are objective and consistent criteria used to define the disease or health issue? ⑤ Have confounding factors been identified? ⑥ Have measures been taken to control confounding factors? ⑦ Are the measurement methods for outcome indicators reliable and valid? ⑧ Is the data analysis method appropriate? Y (Yes): The criterion was met. N (No): The criterion was not met. U (Unclear): The criterion was unclear or not well addressed. NA (Not Applicable): The criterion was not applicable to the study.

### 3.3 Meta-analysis results

#### 3.3.1 Prevalence of musculoskeletal complications

Thirteen studies in this review assessed muscle pain among operating room personnel, with an overall prevalence rate of 56.52% [95%*CI* (47.64, 65.03%), *I*^2^ = 76%]. Heterogeneity was primarily driven by the study by Simonsen et al. (35). After excluding this study, the prevalence rate increased to 60.79% [95%*CI* (52.83, 66.67%), *I*^2^ = 54%]. Among these, nine studies specifically addressed low back pain, with a prevalence rate of 76.13% [95% *CI* (65.93, 86.65%), *I*^2^ = 86%]. Sequential exclusion analysis revealed that after removing the studies by Bos et al. (40), Sheikhza et al. (39) and Choobine et al. (38), the prevalence rate of low back pain was 69.42% [95% *CI* (66.82, 72.51%), *I*^2^ = 1%]. Given the reduced heterogeneity, a fixed-effect model was adopted for analysis. For shoulder and neck pain, nine studies reported a prevalence rate of 63.14% [95% *CI* (57.93, 73.89%), *I*^2^ = 93%], indicating substantial heterogeneity. Subgroup analysis showed that after excluding studies published before 2010, the prevalence rate was 62.71% [95% *CI* (59.87, 66.15%), *I*^2^ = 25%]. Five studies addressed knee pain, reporting a prevalence rate of 58.91% [95% *CI* (53.74, 70.27%), *I*^2^ = 96%]. Due to the small number of included studies, subgroup analysis for heterogeneity was not feasible, and a random-effects model was employed for the analysis.

#### 3.3.2 Prevalence of job burnout

**Figure 2:**
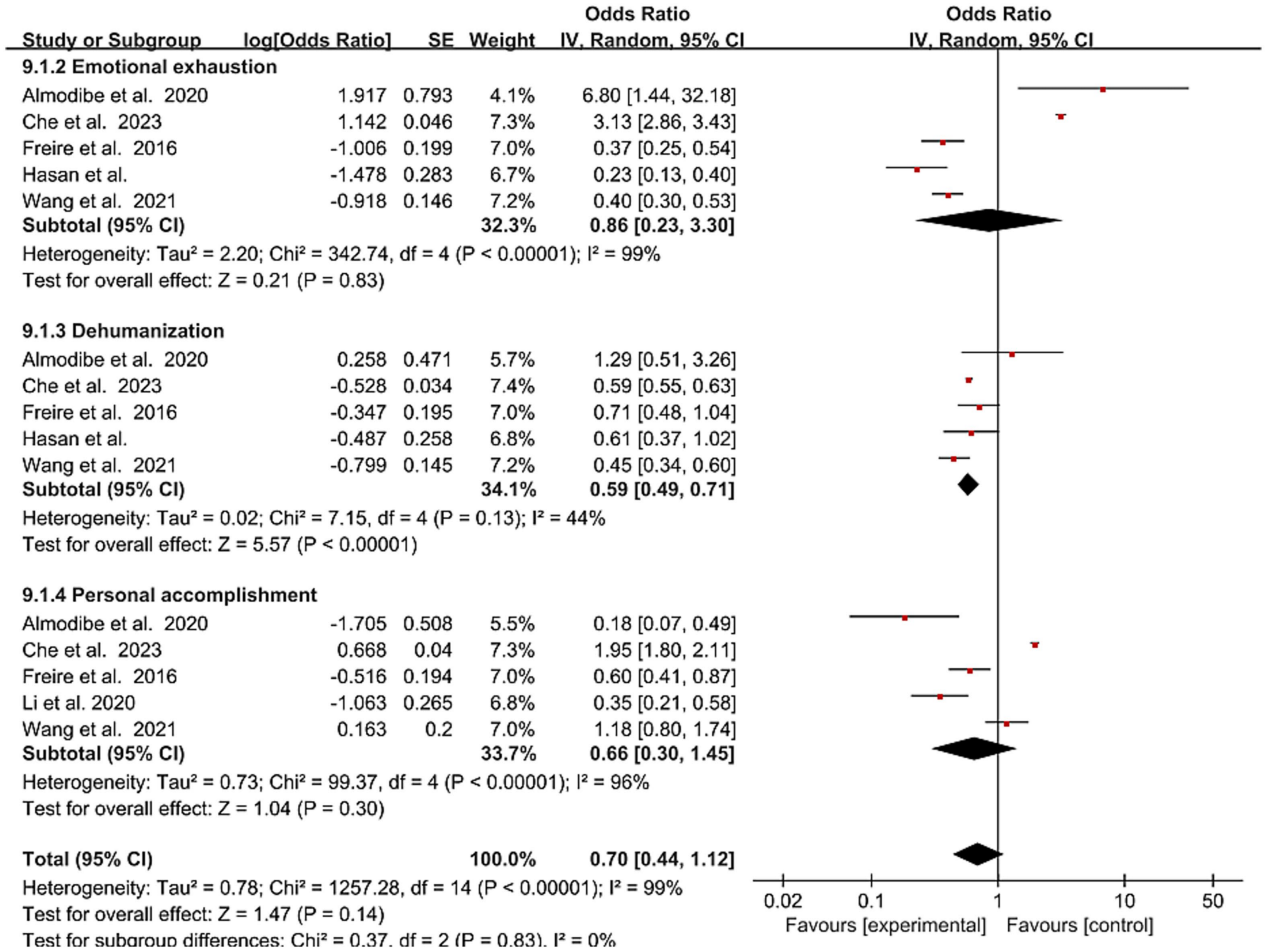
Forest plot of job burnout prevalence across three dimensions (emotional exhaustion, depersonalization, personal accomplishment).

This study included six studies examining occupational burnout among operating room nurses, all of which utilized the Maslach Burnout Inventory (MBI) with dimensions of emotional exhaustion, depersonalization, and reduced personal accomplishment. The overall burnout prevalence across these studies was 52.38% [95% *CI* (42.86, 65.15%), *I*^2^ = 88%], indicating high heterogeneity. Therefore, a random-effects model was employed for the analysis. After excluding two studies that focused on operating room nurses, the burnout prevalence among anesthesiologists was found to be 53.05% [95% *CI* (50.00, 56.14%), *I*^2^ = 20%]. Regarding the three burnout dimensions, emotional exhaustion had a prevalence of 46.24% [95% *CI* (18.70, 76.74%), *I*^2^ = 99%], depersonalization had a prevalence of 44% [95% *CI* (22.21, 67.76%), *I*^2^ = 99%], and reduced personal accomplishment was 39.76% [95% *CI* (23.08, 59.18%), *I*^2^ = 99%] (Figure 2). Due to the high heterogeneity in these results, a random-effects model was also used for these analyses. Li et al. (10)stratified burnout by age among operating room nurses, reporting a cumulative burden of emotional exhaustion that was positively correlated with age. However, the large-sample studies by Almodibeg and Smith (26) and Che et al. (19) disproportionately influenced the overall results due to extreme sample size imbalances. These large studies carried disproportionately high weights with extremely small standard errors, which made the *I*^2^ index highly sensitive to minor biases in effect sizes, thus artificially inflating the perceived statistical heterogeneity.

#### 3.3.3 Prevalence of fatigue

Due to the lack of heterogeneity in fatigue-related literature assessment criteria and outcome reporting, this study conducted only a descriptive analysis. Zhai et al. (13) assessed nurse fatigue using the Occupational Fatigue Exhaustion Recovery scale, Alotaibi et al. (17) evaluated fatigue among operating room healthcare personnel using the FSS scale, and Rambod et al. (16) assessed nurse fatigue using the MFI scale. Results indicated that regardless of the assessment tool used, operating room personnel exhibited high levels of fatigue. Additionally, Rahmani et al. (12) assessed mental fatigue, revealing that 76.88% of nurses experienced mental fatigue. Fu et al. (9) evaluated compassion fatigue among nurses, finding that 25% of nurses exhibited compassion fatigue.

#### 3.3.4 Prevalence of occupational exposure

Operating room personnel are a high-risk group for exposure to bloodborne pathogens. This study included five investigations examining blood and body fluid exposure among operating room nurses, with an overall prevalence rate of 45.05% [95% CI (27.54, 63.90%), I^2^ = 98%], indicating substantial heterogeneity. Three studies reported a sharp injury prevalence of 16.07%, while two studies indicated a needle stick injury prevalence of 25.51%. Two studies assessed exposure to surgical smoke, revealing a prevalence rate of 69.71%. Additionally, three studies reported latex allergies, with a prevalence of 10.65%. Kumari et al. (42) analyzed radiation doses received by operating room personnel on their fingers and torsos, finding varying doses across roles, all of which remained within safe limits. Uğurlu et al. (31) reported exposure to infectious waste at 25%, chemotherapy drug exposure at 47%, and contact dermatitis at 50%. However, these findings should be interpreted with caution due to the small sample sizes, which may limit the generalizability and require further validation.

#### 3.3.5 Prevalence of mental disorders

This study included four literature reviews assessing anxiety among operating room personnel, utilizing tools such as the GAD-7, HADS, and BAI scales. The overall prevalence of anxiety was found to be 36.31% [95% *CI* (9.91, 75.06%), *I*^2^ = 100%], indicating substantial heterogeneity, which necessitated the use of a random-effects model for analysis. This variation may be attributed to differences in the anxiety assessment scales used across studies. Additionally, the study by Che et al. (19) involved a large sample size, potentially introducing a “precision trap,” where observed differences may not be explained by sampling error. Five studies reported on depression among operating room medical personnel, using scales such as the BDI, PHQ-9, and HADS. The overall prevalence of depression was 59.14% [95% *CI* (54.24, 68.78%), *I*^2^ = 98%], again showing substantial heterogeneity, which required a random-effects model for analysis. Che et al. (19) reported a 21.81% prevalence of post-traumatic stress disorder (PTSD). Kim and Shin (11), using the IES-R assessment, found an average score of 1.58 ± 0.9 (out of 4) for PTSD symptoms. Wang et al. (24) used a self-report questionnaire to assess work-related traumatic events, revealing a prevalence of 42.94%, though the small sample size of this study warrants further research for validation. Freire et al. (28) used the Rosenberg Self-Esteem Scale to evaluate low self-esteem among anesthesiologists, reporting a prevalence of 34.2%. Additionally, Yao et al. (15) found a 79.1% prevalence of obsessive-compulsive disorder (OCD) among operating room nurses and a 56.72% prevalence of neurasthenia.

## 4 Discussion

This study systematically assessed the multiple health risks faced by operating room personnel, revealing that this group is at high risk for musculoskeletal issues, mental health concerns, occupational burnout, and environmental exposures. The overall prevalence of muscle pain among operating room personnel was 56.52%, with low back pain (76.13%) and shoulder-neck pain (63.14%) being the most prominent. These findings confirm the operating room as a high-risk environment for cumulative occupational trauma and highlight position-specific injury mechanisms. The musculoskeletal issues observed are likely associated with cumulative damage to spinal health caused by prolonged static loads and forced postures unique to the operating room. For example, surgeons and scrub nurses often experience forward neck flexion, anesthesiologists frequently adopt bending postures during induction, and all personnel engage in high-intensity physical activities such as patient handling and operating heavy instruments (46). Excluding the study by Simoska et al. (47) significantly reduced heterogeneity, suggesting regional or specialty-based variations in ergonomic conditions within operating rooms. Additionally, the prevalence of shoulder and neck pain showed reduced heterogeneity in studies conducted after 2010, which may correlate with the increased use of minimally invasive and endoscopic surgeries. Surgeons performing these procedures often maintain prolonged screen fixation and instrument manipulation, leading to static muscle loading that accelerates wear and tear on the cervical spine and surrounding soft tissues (48).

This study found a high prevalence of depression (59.14%) and anxiety (36.31%) among operating room personnel, with an especially elevated rate of OCD that significantly exceeded that observed in the general population and nurses working in regular wards. This heightened prevalence of OCD is often linked to the deep internalization of stringent sterile protocols and the zero-tolerance safety culture ingrained in the psychological landscape of healthcare workers. The overall burnout rate was 52.38%, with anesthesiologists experiencing slightly higher levels of burnout compared to nurses. This may be related to the intense concentration required by anesthesiologists to monitor vital signs during surgery, coupled with the significant pressure from managing unexpected risks.

Among the burnout dimensions, emotional exhaustion (46.24%) exhibited the greatest variability, with a 95% *CI* (18.70% ~ 76.74%). Li et al. (10) found a positive correlation between emotional exhaustion and age, indicating that psychological resources tend to be irreversibly depleted as healthcare professionals progress in their careers. As clinical experience increases, individuals face not only greater clinical stress but also additional managerial and teaching responsibilities, which further accelerate the depletion of emotional resources. The extreme heterogeneity observed in the analyses of anxiety and burnout (*I*^2^ = 88% ~ 100%) can be attributed not only to differences in assessment tools (e.g., GAD-7 vs. HADS) but also to the “high precision trap” created by large-scale studies like those by Almodibeg and Smith (26) and Che et al. (19). These studies, with their extremely small standard errors, make statistical tests highly sensitive to minute differences in effect sizes across studies, creating an illusion of high heterogeneity. This phenomenon, however, accurately reflects the complex and multifaceted stressors faced by operating room personnel across diverse medical cultures.

Descriptive analysis reveals that operating room personnel consistently experience high levels of fatigue, regardless of the measurement scale used. Among these, mental fatigue (76.88%) significantly surpasses physical fatigue. Mental fatigue directly impacts attention allocation and reaction speed during surgery, which poses a potential risk for medical errors. Of particular concern is compassion fatigue, with one-quarter of healthcare workers reporting diminished empathy due to prolonged exposure to patient suffering and life-and-death decisions. This emotional numbness not only undermines the humanistic quality of care but also serves as a precursor to psychological breakdown. These findings highlight the need for hospitals to address both physical and mental fatigue by implementing strategies such as shift rotation to alleviate physical strain and establishing psychological intervention mechanisms to support the emotional well-being of healthcare workers (49).

Exposure to blood and bodily fluids, along with needle stick injuries, remain traditional biosafety risks for operating room personnel. Despite the widespread adoption of modern infection control techniques, the high intensity of intraoperative coordination makes sharp injuries difficult to prevent. Although the prevalence rate of needle stick injuries has decreased compared to previous years, it remains the primary route for the transmission of bloodborne pathogens. Additionally, the high prevalence of latex allergies and contact dermatitis reflects the chronic damage to the skin barrier caused by prolonged use of intraoperative protective equipment.

This study found that exposure to surgical smoke far exceeds the prevalence of needle stick injuries. Surgical smoke contains hydrocarbons, hydrogen cyanide, and other bioactive substances, which have carcinogenic and pro-inflammatory effects. The cumulative impact of long-term exposure to these substances has been severely underestimated in previous occupational protection measures (50). Furthermore, the 47% exposure rate to chemotherapy drugs underscores the urgent need to enhance occupational protection for personnel involved in procedures such as intraperitoneal hyperthermic chemotherapy. While Kumari et al. (42) concluded that radiation exposure remains within safe limits, this study emphasizes the need to focus on the cumulative dose effects, especially in light of the increasing prevalence of interventional procedures.

In addition to the previously discussed health risks, reproductive health concerns present an invisible yet significant threat to operating room personnel. Prolonged exposure to trace anesthetic gasses, particularly during the induction and recovery phases, has been linked to increased risks of infertility, spontaneous abortion, and fetal malformations (51). Moreover, metabolic syndrome, which results from disrupted circadian rhythms, and the “secondary victim syndrome” triggered by adverse medical events, also warrant attention (52).

In today’s era of high-tech medical interventions, healthcare workers face novel cognitive demands, often stemming from technical pressures. These demands interact with physical injuries and occupational burnout, further exacerbating the health risks experienced by operating room personnel. The Operating Room Occupational Health Cluster proposed in this study is not merely a collection of individual injuries, but a vicious cycle of mutual causation. Chronic musculoskeletal pain lowers an individual’s threshold for stress, triggering anxiety and depression. In turn, severe mental exhaustion diminishes procedural precision, increasing the risks of occupational exposure and needle stick injuries. This multidimensional imbalance ultimately contributes to talent attrition and compromises the overall quality of healthcare.

## 5 Limitation

This study has several limitations. First, there was non-uniformity in diagnostic criteria across the included studies, with some utilizing self-report scales rather than clinical gold standards. This could introduce reporting bias, potentially overestimating or underestimating prevalence rates. Additionally, a few large-sample studies were given disproportionately high weight in the meta-analyses, which may obscure the specific characteristics of smaller, high-quality studies. Furthermore, most of the included studies were cross-sectional, limiting the ability to establish longitudinal causal relationships between occupational exposure and health outcomes. Lastly, raw data for certain indicators were concentrated in specific countries, raising concerns about the global representativeness of the findings.

## 6 Conclusion

The health status of operating room personnel is concerning, characterized by multidimensional harm. The high prevalence of occupational exposure and psychological disorders is not primarily due to inadequate personal protection or insufficient stress resilience, but rather the high-pressure environment of the operating room and systemic deficiencies in protective measures. Hospital administrators should implement targeted interventions, such as optimizing ergonomic designs to reduce musculoskeletal injuries, offering psychological counseling to alleviate occupational burnout, and enhancing operating room exhaust systems and protective equipment to mitigate environmental risks. Future research should focus on establishing unified assessment standards and exploring long-term intervention mechanisms to improve the overall health of operating room personnel.

## Data Availability

The original contributions presented in the study are included in the article/supplementary material, further inquiries can be directed to the corresponding author.
